# To Contributors

**Published:** 1871-11

**Authors:** 


### EDITORIAL AND MISCELLANEOUS.

### TO CONTRIBUTORS.

It is regarded as proper to state, that while it is our intention to publish communications (as intimated in the last number) in the order in which they are received, this rule must necessarily be subject to some modification. It is our object to have some regard to a distribution, as far as practicable, of the original matter among the contributors from the various sections of the United States, and also to have reference to a diversity in the subjects selected for publication in each number. These conditions, in some instances, may postpone the publication of articles which may be upon hand; and in all cases where there is delay, we desire it to be understood that there is some special reason having reference to them, or other important considerations controlling the conduct of a journol which proposes, in some degree, to be the representative of the Medica[ profession of the entire country. Delay, therefore, in publication will be no evidence of a want of appreciation of the value of a contribution; for in each case where an article from any cause is regarded as unsuited to our columns, the author will be promptly informed of the fact.

In this connection, we desire to express our thanks for the manifestations of interest in the success of the Journal in its renewed effort to do a higher work for the profession, as contained in the various communications received from a number of prominent medical men throughout a widely-extended territory.

